# A novel in vitro assay to study chondrocyte-to-osteoblast transdifferentiation

**DOI:** 10.1007/s12020-021-02853-4

**Published:** 2021-09-16

**Authors:** Miriam E. A. Tschaffon, Stefan O. Reber, Astrid Schoppa, Sayantan Nandi, Ion C. Cirstea, Attila Aszodi, Anita Ignatius, Melanie Haffner-Luntzer

**Affiliations:** 1 Institute of Orthopedic Research and Biomechanics, University Medical Center Ulm, Ulm, Germany; 2 Laboratory for Molecular Psychosomatics, Department of Psychosomatic Medicine and Psychotherapy, University of Ulm, Ulm, Germany; 3 Institute of Comparative Molecular Endocrinology, University of Ulm, Ulm, Germany; 4 Laboratory of Experimental Surgery and Regenerative Medicine, Clinic for General, Trauma and Reconstructive Surgery, Klinikum der Universität München, Martinsried, Germany

**Keywords:** Transdifferentiation, Cartilage to bone transformation, Endochondral ossification, Chondrocyte, In vitro assay, Fracture healing

## Abstract

**Purpose:**

Endochondral ossification, which involves transdifferentiation of chondrocytes into osteoblasts, is an important process involved in the development and postnatal growth of most vertebrate bones as well as in bone fracture healing. To study the basic molecular mechanisms of this process, a robust and easy-to-use in vitro model is desirable. Therefore, we aimed to develop a standardized in vitro assay for the transdifferentiation of chondrogenic cells towards the osteogenic lineage.

**Methods:**

Murine chondrogenic ATDC5 cells were differentiated into the chondrogenic lineage for seven days and subsequently differentiated towards the osteogenic direction. Gene expression analysis of pluripotency, as well as chondrogenic and osteogenic markers, cell–matrix staining, and immunofluorescent staining, were performed to assess the differentiation. In addition, the effects of Wnt3a and lipopolysaccharides (LPS) on the transdifferentiation were tested by their addition to the osteogenic differentiation medium.

**Results:**

Following osteogenic differentiation, chondrogenically pe-differentiated cells displayed the expression of pluripotency and osteogenic marker genes as well as alkaline phosphatase activity and a mineralized matrix. Co-expression of Col2a1 and Col1a1 after one day of osteogenic differentiation indicated that osteogenic cells had differentiated from chondrogenic cells. Wnt3a increased and LPS decreased transdifferentiation towards the osteogenic lineage.

**Conclusion:**

We successfully established a rapid, standardized in vitro assay for the transdifferentiation of chondrogenic cells into osteogenic cells, which is suitable for testing the effects of different compounds on this cellular process.

## Introduction

Most parts of the vertebrate skeleton, including vertebrae, ribs, and long bones, form through endochondral ossification. In this process, bone tissue is generated through a cartilage intermediate formed by chondrocytes. This cartilage scaffold is progressively replaced by bone during fetal development and postnatal growth [1]. A small cartilaginous region, the growth plate, remains in the epiphyseal region of long bones for as long as bone growth continues. Within the growth plate, chondrocytes are arranged in columns parallel to the long axis of the bone [2]. Growth plate chondrocytes proliferate, then differentiate into prehypertrophic and thereupon into hypertrophic chondrocytes. When chondrocytes enter the proliferative state, they start to synthesize proteoglycans, including aggrecan (encoded by the *Acan* gene) and different types of collagen, for example, collagen type II (*Col2a1*) [3, 4]. When growth plate chondrocytes present as hypertrophic cells, they express collagen type X (*Col10a1*) [2, 5], whereas collagen type II synthesis decreases in hypertrophic chondrocytes [6]. The produced cartilage serves as a scaffold for the bone matrix produced by osteoblasts. Osteoblasts deposit osteoid, a collagen-proteoglycan-rich matrix, which is then mineralized by the same cells [7]. Mature osteoblasts develop from osteoblast precursors expressing Runt-related transcription factor 2 (Runx2, encoded by the *Cbfa1* gene), which regulates the expression of other osteoblast marker genes, including Osterix (*Sp7*), bone matrix proteins such as collagen type I and bone sialoprotein (*Ibsp*) as well as alkaline phosphatase (ALP, encoded by the *Alpl* gene), which is important for mineralization [8–10]. During secondary bone healing, which involves the formation of a cartilaginous fracture callus, a similar mechanism to that found in the growth plate during endochondral ossification occurs when cartilaginous tissue in the callus is replaced by bony tissue [11, 12].

For decades it was thought that all terminally differentiated hypertrophic chondrocytes in the cartilage-to-bone transition zone undergo apoptosis or autophagy and new bone is exclusively formed by osteoblasts, which differentiate from invading mesenchymal progenitors [13–15]. However, recent studies using lineage-tracing mouse models demonstrated that numerous hypertrophic chondrocytes at the transition zone are able to transdifferentiate into bone-forming osteoblasts, thereby directly contributing to the bone formation during bone development and postnatal growth as well as during bone fracture healing [16–19]. Because this cartilage-to-bone transition appears to be essential for long bone growth and fracture healing, it is important to gain an insight into the molecular mechanisms involved in this process. For rapid, basic investigation of the effects of different compounds on biological processes, in vitro systems are the method of choice. However, so far there are no suitable in vitro models for chondrocyte-to-osteoblast transdifferentiation are available. Therefore, we aimed to develop a standardized, easy-to-use in vitro assay for the transdifferentiation of chondrogenic cells into osteogenic cells using the murine chondrogenic cell line ATDC5. We further aimed to prove the translational relevance of this assay by testing different compounds which are known to accelerate or inhibit chondrocyte-to-osteoblast transdifferentiation in vivo.

## Materials and methods

### Cell culture

**Fig. 1:**
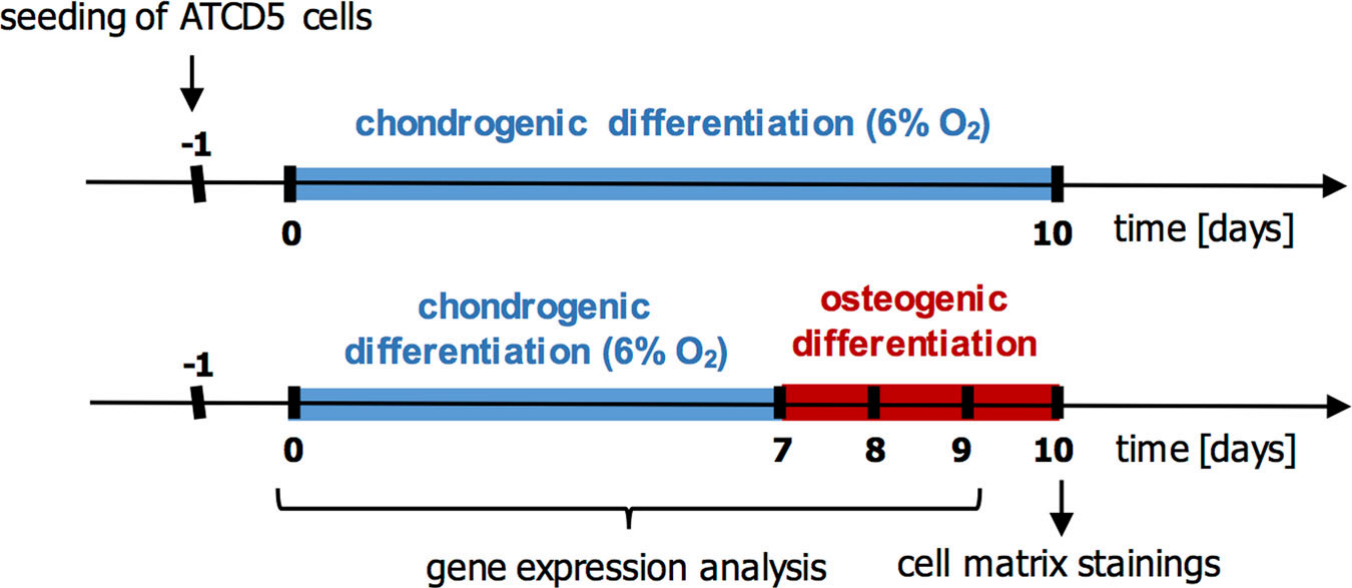
Timeline of cultivation of ATDC5 cells in chondrogenic and osteogenic differentiation media

ATDC5 cells were cultivated at 37 °C in DMEM/F12 (1:1) (Gibco), supplemented with 5% fetal calf serum (FCS) (Merck Millipore), 1% L-glutamine (Gibco), 1% penicillin/streptomycin (Gibco), human transferrin (10 µg/ml, Sigma) and sodium selenite (30 nM). Cells were seeded on 24-well plates at a density of 4000 cells/cm^2^. Cells were differentiated towards the chondrogenic direction for 7 days by adding human insulin (10 µg/ml, Sigma) and ascorbate 2-phosphate (0.2 mM, Sigma) to the medium and changing the oxygen level to 6% during the differentiation time period (until day 7). To induce transdifferentiation, chondrogenic differentiation medium was changed to α-MEM (Biochrom), containing 10% FCS, 1% l-glutamine, 1% penicillin/streptomycin, β-glycerol phosphate (10 mM, Sigma), ascorbate 2-phosphate (0.2 M), and human bone morphogenic protein 2 (BMP-2) (100 ng/ml, Thermo Fisher Scientific) and further cultured for up to 3 days at physiological oxygen concentration. Cells were harvested at days 0 and 7 of chondrogenic differentiation and after 1 and 2 days of osteogenic differentiation to analyze differentiation marker genes as well as after 3 days of osteogenic differentiation for cell–matrix staining. Control cells were cultivated in a chondrogenic differentiation medium for the entire culture period and harvested for cell–matrix staining after 10 days of chondrogenic differentiation (Fig. 1). To investigate the effects of canonical Wnt signaling and lipopolysaccharides (LPS) on the transdifferentiation, Wnt3a (3 nM, R&D Systems) and LPS (0.1 µg/ml, Sigma) was added to the osteogenic differentiation medium, respectively.

### Cell matrix and mineralization staining

Alcian blue staining was performed to examine chondrogenic matrix production. Fixed cells were acidified with 3% acetic acid and subsequently stained with 1% Alcian blue (Sigma) for 30 min. To assess osteoblast activity and matrix mineralization, ALP and von Kossa staining were performed. ALP staining was performed using the Leukocyte Alkaline Phosphatase Kit (Sigma) according to the manufacturer’s instructions. For von Kossa staining, cells were incubated for 50 min with 5% silver nitrate, followed by incubation with 80 mM pyrogallol for 8 min and fixation with 0.2 M sodium thiosulfate pentahydrate solution.

### Gene expression analysis

Total RNA was isolated from the cells using the RNeasy Mini Kit (Qiagen), and each sample was treated with DNase (Qiagen). One-step semi-quantitative Real-Time-PCRs were performed using the SensiFAST SYBR Hi-ROX One-Step Kit (Bioline) and the Real-Time PCR System QuantStudio 3 (Thermo Fisher Scientific). Relative gene expression was calculated by normalizing to the housekeeping gene *B2M* and to expressions of day 0 or day 7 with the ΔΔCT method. Gene expression from experiments with Wnt3a and LPS were normalized to the respective controls of day 8 and day 9. Used primer sequences are listed in Table 1.

**Table 1:** Primer sequences

Gene name	Forward primer sequence (5′ to 3′)	Reverse primer sequence (3′ to 5′)
*Acan*	AACTTCTTTGCCACCGGAGA	GGTGCCCTTTTTACACGTGAA
*Alpl*	GCTGATCATTCCCACGTTTT	GAGCCAGACCAAAGATGGAG
*B2m*	ATACGCCTGCAGAGTTAAGCA	TCACATGTCTCGATCCCAGT
*Bax*	GCTGGACACTGGACTTCCTC	GAGGCCTTCCCAGCCAC
*Bcl-2*	AGTACCTGAACCGGCATCTG	GGGGCCATATAGTTCCACAAA
*Cbfa1*	CCACCACTCACTACCACACG	CACTCTGGCTTTGGGAAGAG
*Col2a1*	CCTGTCTGCTTCTTGTAAAAC	TGGGTATCATCAGGTCAGGT
*Col10a1*	CATCTCCCAGCACCAGAATC	CCCATGAACCAGGGTCAAGAA
*Ibsp*	GAAGCAGGTGCAGAAGGAAC	GAAACCCGTTCAGAAGGACA
*Nanog*	AAGGATGAAGTGCAAGCGGT	GGTGCTGAGCCCTTCTGAAT
*Sox2*	CAAAAACCGTGATGCCGACT	CGCCCTCAGGTTTTCTCTGT
*Sp7*	CCTTAACCCAGCTCCCTACC	ACCGCCTTGGGCTTATAGAC

### Live cell imaging

On day 7 after chondrogenic differentiation, live-cell imaging of cells was performed directly after transferring the cells into osteogenic differentiation medium using microscope Leica DMI6000B at 37 °C with 5% CO_2_. Imaging was performed for 24 h taking an image every 15 min.

### Immunofluorescent staining

Cells were fixed with 4% formalin after 7 days of chondrogenic differentiation or after 1 or 3 days of subsequent osteogenic differentiation. Cells were blocked with 4% bovine serum albumin (Sigma) and immunofluorescent staining was performed using the primary antibodies rabbit anti-Col2a1 (Rockland, 1:100) and goat anti-Col1a1 (Santa Cruz, 1:100) overnight at 4 °C and secondary antibodies AF594 donkey anti-rabbit (Invitrogen, 1:250) and biotinylated donkey anti-goat antibody (Santa Cruz, 1:200) for one hour at room temperature (RT), respectively, followed by incubation with FITC-Streptavidin (Biolegend, 1:200) for 30 min at RT. Host-specific IgG isotype controls were used as a negative control instead of primary antibodies.

### TUNEL assay

Apoptosis was assessed using the CF^TM^ 488A TUNEL Apoptosis Detection Kit (Biotium). The assay was performed according to the producer’s manual. Briefly, cells were fixed with 4% formalin after 7 days of chondrogenic differentiation or after 1 or 2 days of subsequent osteogenic differentiation. Cells were permeabilized in 0.2% Triton X-100 for 30 min, washed twice with PBS, and incubated for 5 min in TUNEL Equilibration Buffer. The subsequent TUNEL reaction was performed for 1 h at 37 °C, followed by washing with PBS containing 0.1% Triton X-100 and 5 mg/mL bovine serum albumin.

### Statistical analysis

Each experiment was performed at least twice in duplicates or triplicates. The final number of samples is indicated in the respective figure legend. Data are displayed as mean + standard deviation. Statistically significant differences were assessed between the groups by performing the appropriate statistical test using GraphPad Prism 9. The used statistical tests are detailed in the respective figure legends.

## Results

**Fig. 2:**
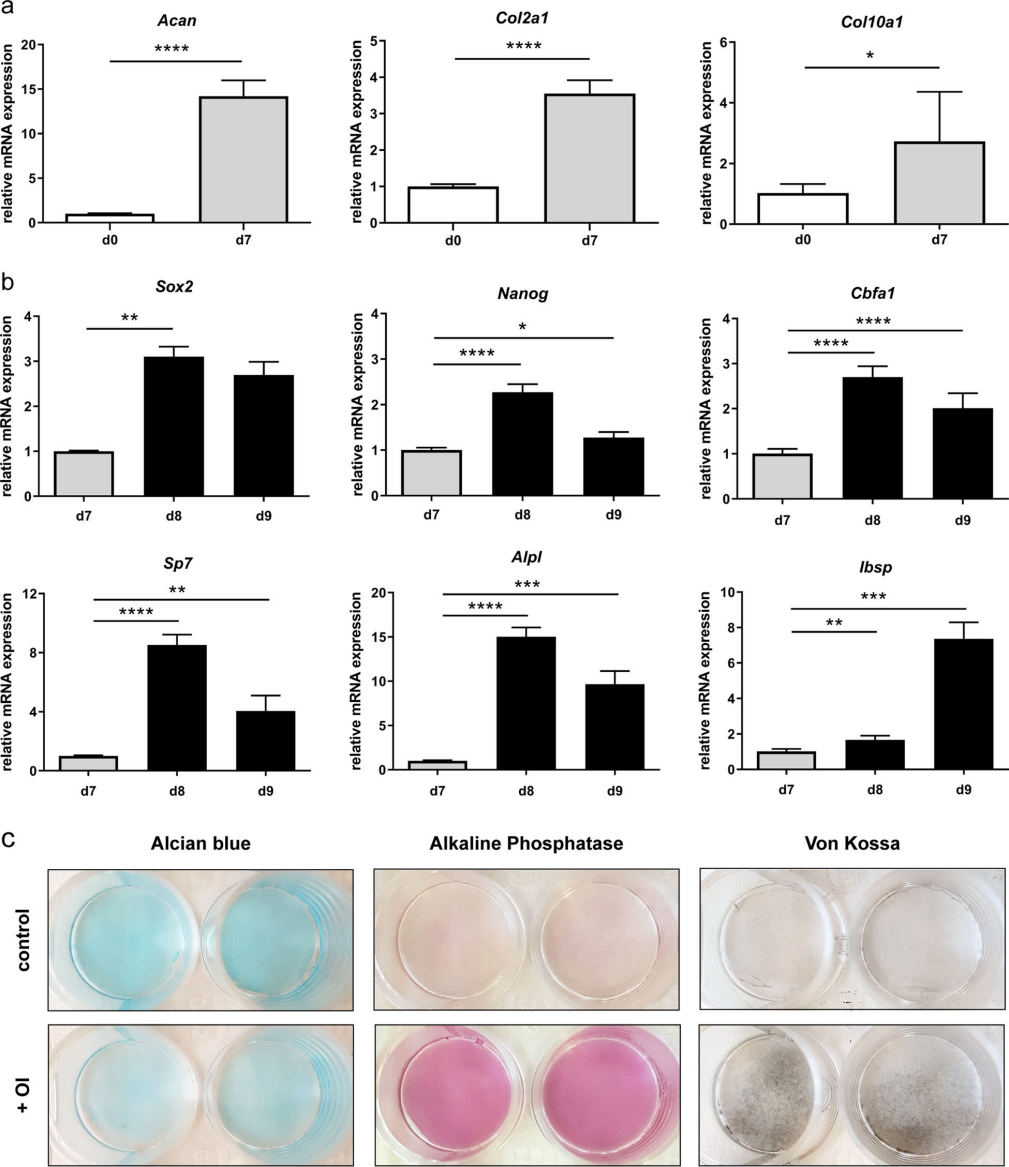
Relative mRNA expression of **a** chondrogenic marker genes before (d0) and after 7 days of chondrogenic differentiation (d7) and **b** pluripotency genes (*Sox2*, *Nanog*) and osteogenic marker genes (*Cbfa1*, *Sp7*, *AlpI*, *Ibsp*) before (d7) and after osteogenic differentiation (d8 and d9). Statistical analysis was performed using Student’s *t* test, one-way-ANOVA or Kruskal–Wallis test. *n* = 5. *p < 0.05; **p < 0.01; ***p < 0.001; ****p < 0.0001. **c** Alcian blue, alkaline phosphatase, and von Kossa staining of cell–matrix after 3 days of osteogenic differentiation (d10). Control cells were incubated in a chondrogenic differentiation medium for all 10 days. *n* = 12. OI osteogenic induction

Several experimental conditions to induce chondrocyte-to-osteoblast transdifferentiation were tested (data not shown). Transdifferentiation was only achieved when the chondrogenic medium was changed to osteogenic medium after 7 days of cultivation, recombinant BMP-2 was added to the osteogenic culture medium and oxygen concentrations were adapted to the different cultivation phases. With this protocol, after 7 days of chondrogenic differentiation, ATDC5 cells displayed significantly upregulated gene expression of *Acan*, *Col2a1*, and *Col10a1* (Fig. 2a). Following one or two days of incubation with osteogenic differentiation medium (d8 and d9 of the experiment), cells upregulated the expression of the pluripotency genes *Sox2* and *Nanog* as well as of the osteogenic marker genes *Cbfa1*, *Sp7*, *Alpl*, and *Ibsp* (Fig. 2b). After 3 days of osteogenic differentiation (d10 of the experiment), cells displayed the production of a cartilage matrix positively stained with Alcian blue, which was less intense compared to control cells incubated in a chondrogenic differentiation medium for 10 days. Furthermore, ALP activity and mineralized matrix were increased in cells cultivated under transdifferentiation conditions (Fig. 2c). No morphological changes were observed between control cells kept in chondrogenic differentiation medium and cells transferred into osteogenic differentiation medium during live-cell imaging of the cells from day 7 to day 8 (see Supplementary videos S1 and S2).

**Fig. 3:**
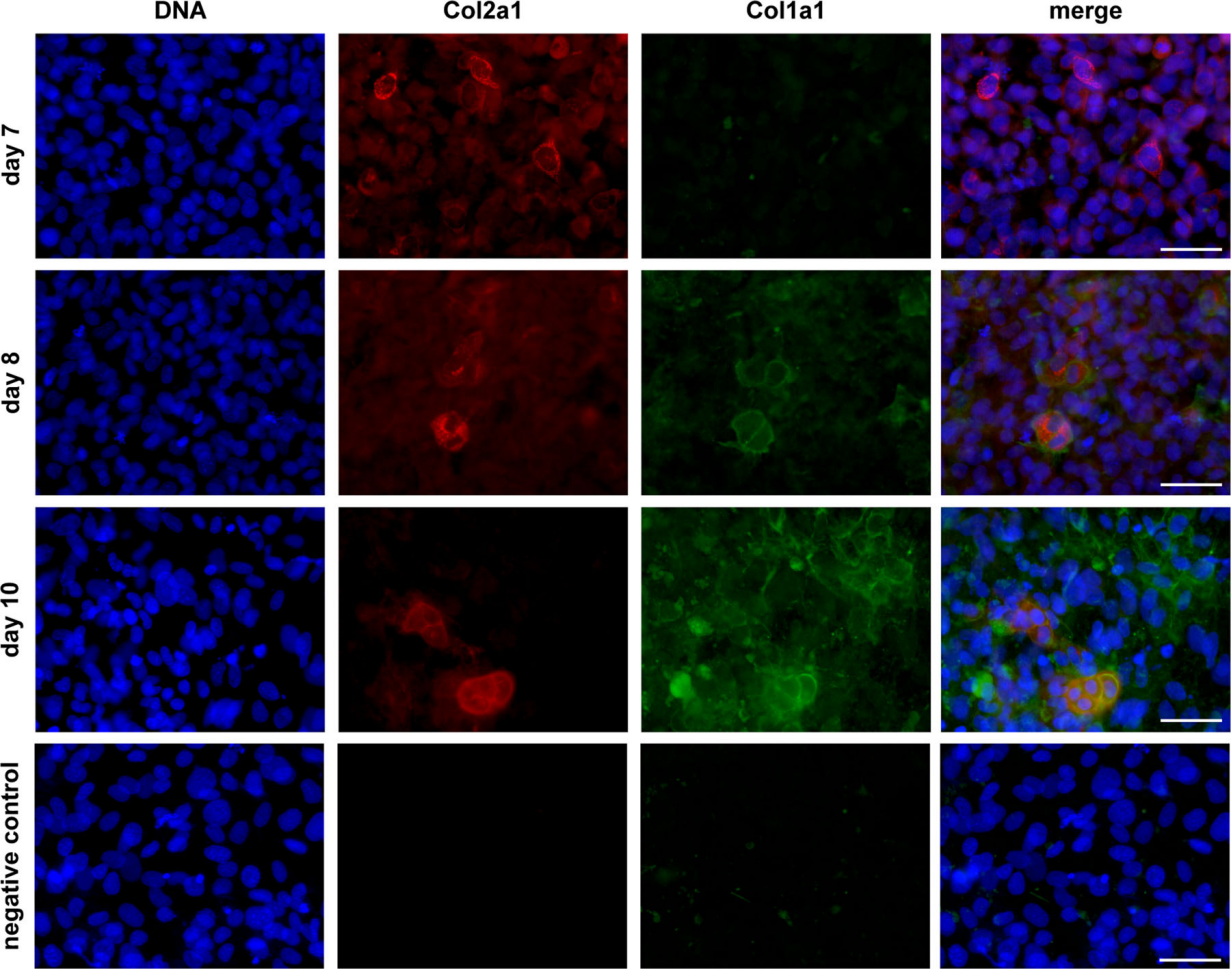
Immunofluorescent detection of Col2a1 and Col1a1 in cells harvested after 7 days of chondrogenic differentiation (day 7) and after 1 or 3 days of osteogenic differentiation (day 8 and 10). Negative control images display cells stained with DAPI and the respective IgG controls. Scale bars represent 50 µm

On day 7 of chondrogenic differentiation, most of the cells displayed positive staining for the chondrogenic marker collagen type II (Col2a1) but not the osteogenic marker collagen type I (Col1a1) (Fig. 3, day 7). This indicated that ATDC5 cells did not differentiate spontaneously into osteoblasts during cell culture under the above-mentioned chondrogenic conditions. After one day of osteogenic differentiation, approximately 10–20% of the cells displayed a strong co-expression of Col2a1 and Col1a1, indicating an ongoing transdifferentiation process (Fig. 3, day 8). After three days of osteogenic differentiation, clearly more cells expressed Col1a1 whereas the number of Col2a1 expressing cells slightly decreased, however co-expression of Col2a1 and Col1a1 was still observed in approximately 10% of the cells (Fig. 3, day 10).

**Fig. 4:**
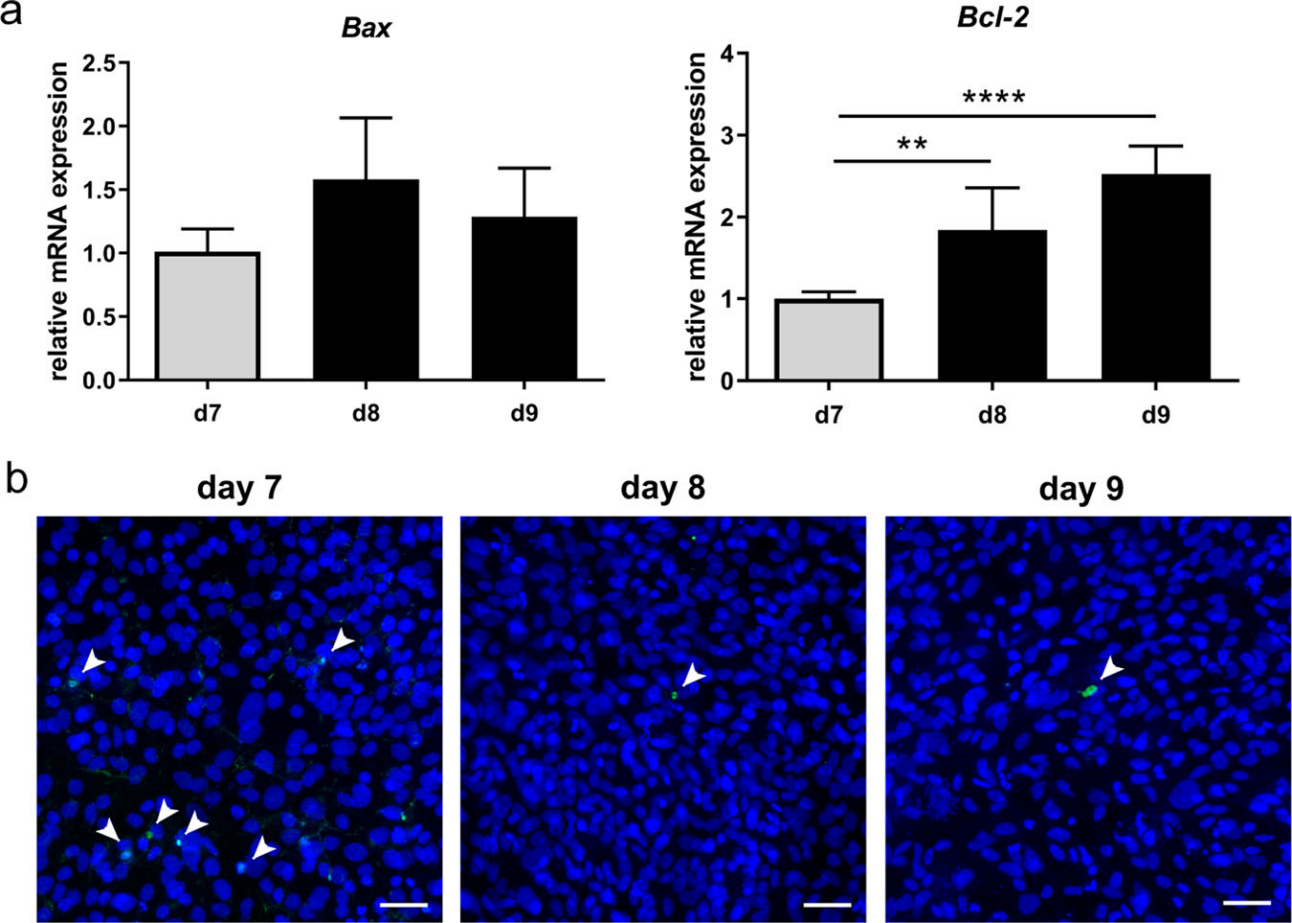
**a** Relative mRNA expression of *Bax* and *Bcl-2*. Statistical analysis was performed using one-way ANOVA. *n* = 5. **p < 0.01; ****p < 0.0001. b TUNEL assay performed on cells harvested after 7 days of chondrogenic differentiation (day 7) and after 1 or 2 days of osteogenic differentiation (day 8 + 9). TUNEL-positive cells are marked by arrowheads. Scale bars represent 50 µm

To compare apoptosis of cells after 7 days of chondrogenic differentiation (day 7) and after 1 or 2 days of osteogenic differentiation (day 8 + 9), gene expression analysis of the apoptosis-regulating factors BCL2-associated X-protein (*Bax*) and B cell leukemia/lymphoma 2 (*Bcl-2*), as well as a TUNEL assay, were performed. Expression of *Bax* did not change whereas *Bcl-2* expression changed 2-fold on day 8 and 2.5-fold on day 9, compared to day 7 (Fig. 4a). In general, only a few TUNEL-positive cells were observed, whereupon more TUNEL-positive cells were observed on day 7 compared to day 8 and 9 (Fig. 4b).

**Fig. 5:**
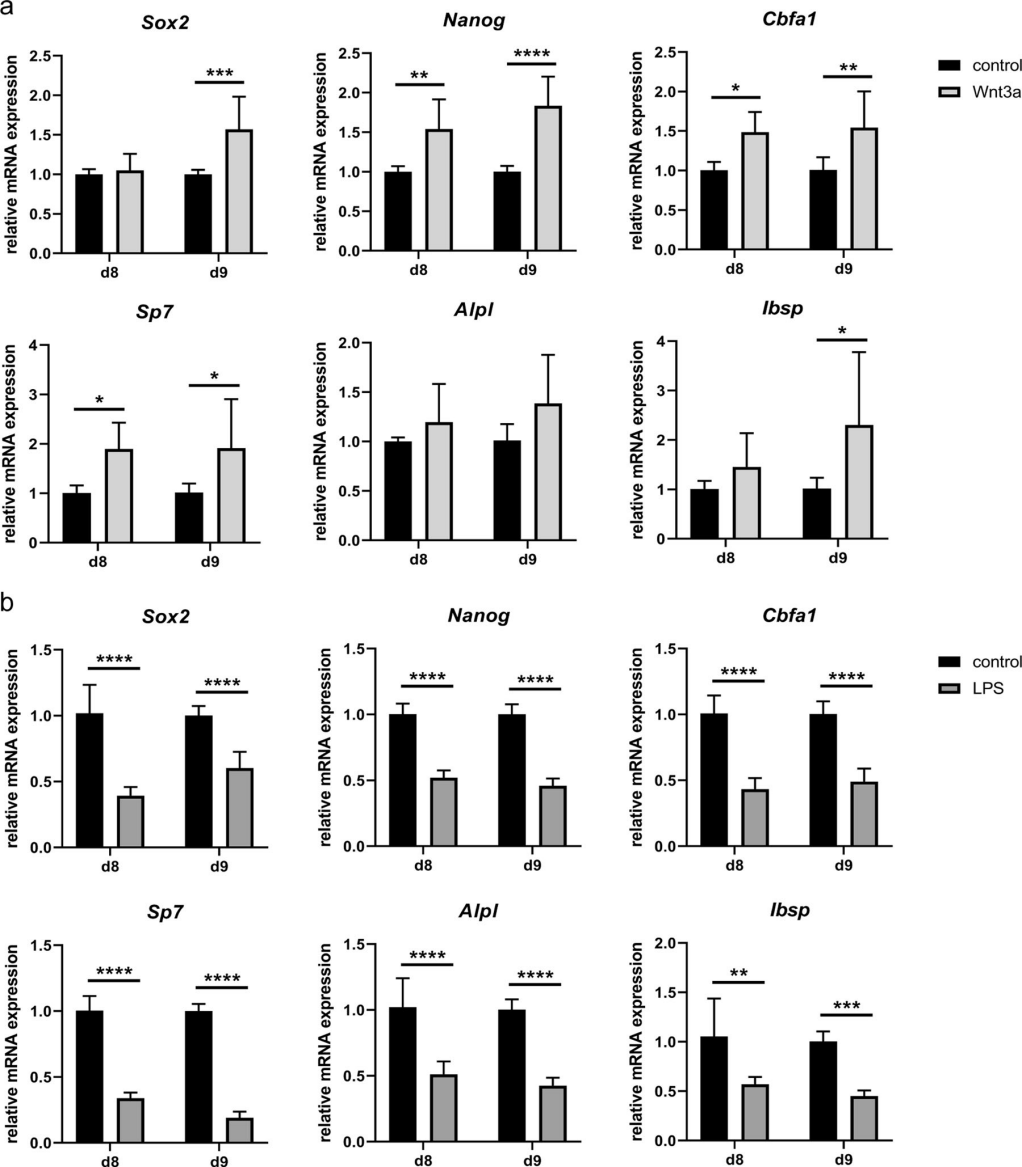
Relative mRNA expression of pluripotency and osteogenic marker genes of transdifferentiating ATDC5 cells treated with **a** 3 nM Wnt3a or **b** 0.1 µg/ml LPS. Statistical analysis was performed using the two-way ANOVA or Mann–Whitney test. *n* = 6. *p < 0.05; **p < 0.01; ***p < 0.001; ****p < 0.0001

The addition of Wnt3a to the osteogenic differentiation medium increased the upregulation of the pluripotency genes *Sox2* and *Nanog* and of the osteogenic marker genes *Cbfa1*, *Sp7*, and *Ibsp* up to twofold (Fig. 5a). This indicates an acceleration of the transdifferentiation process upon activation of the canonical Wnt signaling pathway. By contrast, the addition of LPS reduced the expression of these pluripotency and osteogenic marker genes by approximately 50% (Fig. 5b), indicating a delayed transdifferentiation process.

## Discussion

We successfully established a rapid, standardized, and easy-to-use in vitro chondrocyte-to-osteoblast transdifferentiation assay. In our experimental protocol, the upregulation of chondrogenic marker genes and the production of cartilage matrix indicate chondrogenic differentiation of ATDC5 cells after the first 7 days of the experiment. The threefold upregulation of *Col10a1* expression, which is exclusively expressed by prehypertrophic and hypertrophic chondrocytes [20], indicates the beginning hypertrophy of the cells. This is important for the translational relevance of our assay because, in vivo, it was shown that particularly hypertrophic chondrocytes at the transition zone are able to transdifferentiate into bone-forming osteoblasts, thereby directly contributing to the bone formation during bone development and postnatal growth as well as during bone fracture healing [16–19]. In our assay, the lower intensity of Alcian blue staining compared to control cells incubated in a chondrogenic differentiation medium for 10 days suggests that the cells did indeed stop or reduce cartilage matrix production after the induction of transdifferentiation.

*Sox2* and *Nanog* upregulation after the induction of osteogenic differentiation is in agreement with a study of Bahney and colleagues which demonstrated the expression of pluripotency genes in hypertrophic chondrocytes in the cartilage-to-bone transition zone of the fracture callus [19]. Upregulation of osteogenic marker genes indicates successful transdifferentiation towards the osteoblast lineage. ALP activity and matrix mineralization indicate that cells have transformed into active, mineralizing osteoblasts. Co-localization of Col2a1 and Col1a1 indicates a simultaneous or subsequent expression of chondrogenic and osteogenic markers, respectively. A stage of co-expression of chondrogenic and osteogenic markers during chondrocyte-to-osteoblast transdifferentiation has already been described in vivo [21], indicating that ATDC5 cells do actually transdifferentiate in our experimental setup. Further experiments using a lineage-tracing approach would be desirable to quantify the rate of transdifferentiation in our assay.

The addition of recombinant BMP-2 to the osteogenic differentiation medium was necessary to induce transdifferentiation towards the osteogenic lineage. Bone morphogenic proteins (BMPs) are in frequent clinical use to induce bone formation for the treatment of non-union fractures and in spinal fusion surgery [22]. BMP signaling occurs via the Smad pathway and promotes osteoblastogenesis by inducing or promoting Runx2 and Osterix expression [23]. Therefore, it is reasonable to assume that it is also important for chondrocyte-to-osteoblast transition.

Compared to day 7, *Bax* expression was unchanged whereas the expression of *Bcl-2* was significantly increased on days 8 and 9. Since Bax promotes apoptosis and Bcl-2 inhibits the Bax-induced promotion of apoptosis [24], this indicates a decreased number of apoptotic cells after induction of transdifferentiation. Decreased numbers of TUNEL-positive cells on days 8 and 9, compared to day 7, confirm a decreased apoptosis rate. In vivo, terminal hypertrophic chondrocytes transdifferentiate or undergo apoptosis during endochondral ossification [25]. Our results indicate that in our assay, cells are more likely to undergo transdifferentiation rather than apoptosis. However, in general, the used immortalized cell line is showing only a few TUNEL-positive cells. Therefore, to analyze transdifferentiation versus apoptosis, the use of primary cells would be more suitable.

To further validate our in vitro assay for its translational relevance to the in vivo situation, we screened the literature for known pathways accelerating or inhibiting transdifferentiation and tested some of these scenarios. The increased transdifferentiation of ATDC5 cells through the addition of Wnt3a, an activator of the Wnt signaling pathway, is in agreement with previous findings of in vivo studies [26, 27], which showed that canonical Wnt signaling is required for endochondral ossification during bone development and fracture healing. Mice lacking active β-catenin, a transcriptional co-factor of the canonical Wnt signaling pathway, in hypertrophic chondrocytes displayed reduced chondrocyte-to-osteoblast transdifferentiation in the long bones [26]. By contrast, mice with hypertrophic chondrocyte-specific stabilization of β-catenin showed increased numbers of chondrocyte-derived osteoblasts [26]. Another study demonstrated that mice lacking β-catenin in chondrocytes display reduced cartilage-to-bone transition during fracture healing and that chondrocyte-specific stabilization of β-catenin accelerates endochondral fracture repair [27]. However, the exact molecular mechanism by which canonical Wnt signaling promotes chondrocyte-to-osteoblast transdifferentiation remains to be determined [28] and is beyond the scope of this study.

Patients suffering from acute or chronic systemic inflammation frequently display disturbed fracture healing [8]. To mimic inflammatory conditions, we treated ATDC5 cells with LPS, which decreased their transdifferentiation. A previous study demonstrated that LPS has inhibitory effects on BMP/Smad signaling through a TLR4-MyD88-NFκB pathway, negatively modulating the osteoinductive capacity of BMP-2 in mouse bone marrow mesenchymal stem cells [29]. A similar process could affect chondrocyte-to-osteoblast transdifferentiation, which occurs during bone fracture healing [18, 19], and could contribute to an impaired cartilage-to-bone transition in LPS-treated rats [30]. Indeed, in our assay, LPS also inhibited the process of transdifferentiation.

In conclusion, our novel in vitro assay provides a standardized and easy model for the rapid basic investigation of the effects of different compounds on chondrocyte-to-osteoblast transdifferentiation. Earlier studies have already provided evidence that avian, murine, and human chondrocytes can transform into osteoblast-like or osteocyte-like cells in vitro and synthesize bone-like matrix, although this was not the purpose of those studies [31–35]. The first systematic in vitro model used for the investigation of transdifferentiation during the endochondral ossification process was developed by Bahney and colleagues. Here, cartilaginous explants from murine tibia fracture calli were cultured in vitro to study chondrocyte-to-osteoblast-transdifferentiation during fracture healing [19, 36]. This assay reflects the in vivo situation much better than our assay, however, serious drawbacks are the need to sacrifice animals and the fact that the fracture callus is a very inhomogeneous tissue, thereby limiting to an extent the standardization of such experiments. Of course, our experimental design also has some limitations. This assay uses an immortalized chondrogenic cell line in two-dimensional in vitro cultivation. Therefore, the in vivo situation of endochondral ossification with chondrocytes in a three-dimensional structure and the influence of other cell types is not exactly mimicked and the assay does not seem to be suitable to analyze transdifferentiation versus apoptosis during endochondral ossification. Even so, our data provide evidence that it is a useful model for the investigation of direct effects on chondrocytes during their transdifferentiation.

## Supplementary information

Supplementary videos S1

Supplementary videos S2
